# Factors associated with intravenous lidocaine in pediatric patients undergoing laparoscopic appendectomy – a retrospective, single-centre experience

**DOI:** 10.1186/s12871-018-0545-1

**Published:** 2018-07-18

**Authors:** Christian P. Both, Jörg Thomas, Philipp K. Bühler, Achim Schmitz, Markus Weiss, Tobias Piegeler

**Affiliations:** 1 Department of Anesthesia, University Children’s Hospital, Steinwiesstrasse 75, CH-8032 Zurich, Switzerland; 2 Department of Anesthesiology and Intensive Care Medicine, University Hospital Leipzig, Liebigstraße 20, 04103 Leipzig, Germany

**Keywords:** Lidocaine, Pediatric anesthesia, Laparoscopic appendectomy, Safety, Emergence delirium, Pain

## Abstract

**Background:**

Due to its potential beneficial effects, intra- and postoperative application of intravenous lidocaine has become increasingly accepted over the last couple of years, e.g. in patients undergoing laparoscopic surgical procedures. Based on its beneficial properties, lidocaine was introduced to the standard of care for all pediatric laparoscopic procedures in our institution in mid-2016. In contrast to adult care, scarce data is available regarding the use of perioperative intravenous lidocaine administration in children undergoing laparoscopic procedures, such as an appendectomy.

**Methods:**

Retrospective analysis of all pediatric patients undergoing laparoscopic appendectomy at the University Children’s Hospital Zurich in 2016. Perioperative data, as recorded in the electronic patient data management system, were evaluated for any signs of systemic lidocaine toxicity (neurological and cardiovascular), behavioral deterioration, as well as for hemodynamic instability. Additionally, the incidence of postoperative nausea and vomiting, administration of pain rescue medication, time to hospital discharge and to first bowel movement, as well as any postoperative complications were recorded. Starting on 01/07/2016, all patients undergoing laparoscopic surgery received intravenous lidocaine (1.5 mg/kg body weight (BW) bolus after induction of anesthesia followed by continuous infusion of 1.5 mg/kgBW/h). These patients were then compared to children without lidocaine administration who had undergone laparoscopic appendectomy between 01/01/2016 and 30/06/2016.

**Results:**

Data of 116 patients was analyzed. Of these, 60 patients received lidocaine. No signs of systemic toxicity, neurologic impairment or circulatory disturbances were noted in any of these patients. A (non-significant) difference in the incidence of emergence delirium was observed (0 cases in the lidocaine group vs. 4 cases in the control group, *p* = 0.05).

**Conclusion:**

This retrospective analysis did not reveal any adverse effects in pediatric patients receiving intravenous lidocaine for laparoscopic appendectomy under general anesthesia. However, further trials investigating beneficial effects as well as pharmacokinetic properties of intravenous lidocaine in children are required.

## Background

Lidocaine has been in clinical use for loco-regional anesthesia as well as for the treatment of arrhythmias for several decades [1]. Over the last couple of years, the intravenous application of the drug has also gained more and more attention, e.g. as part of a multimodal therapy concept for perioperative pain [2, 3], although it is still considered to be an off-label use [4].

In adults, several clinical trials have examined potential beneficial effects of perioperative intravenous lidocaine use due to its anti-inflammatory characteristics [5–8], e.g. in patients undergoing laparoscopic procedures [9–13]. A recent Cochrane analysis assessing 45 trials with 2802 mixed surgical patients found low to moderate evidence that intravenous lidocaine might be able to reduce early postoperative pain scores and PONV, as well as some limited evidence for a beneficial effect on other postoperative parameters, such as post-operative opioid requirements, the length of hospital stay or the time to first bowel movement [14]. However, to our knowledge, no data is available regarding the safety of continuous perioperative lidocaine infusion in children.

Based on its potential beneficial effects regarding postoperative pain control or postoperative nausea and vomiting (PONV), we introduced the additive perioperative administration of intravenous lidocaine to our standard anesthesia regimen for all laparoscopic procedures on July 1st, 2016.

The primary goal of this study was to retrospectively evaluate adverse effects of the intra- and post-operative application of intravenous lidocaine in patients undergoing laparoscopic appendectomy compared to a historical cohort prior to the introduction of lidocaine. Additionally, the study aimed at evaluating any potential beneficial effects of intravenous lidocaine, such as postoperative opioid requirements, PONV, time to hospital discharge and bowel movement as well as any postoperative complications.

## Methods

**Fig. 1:**
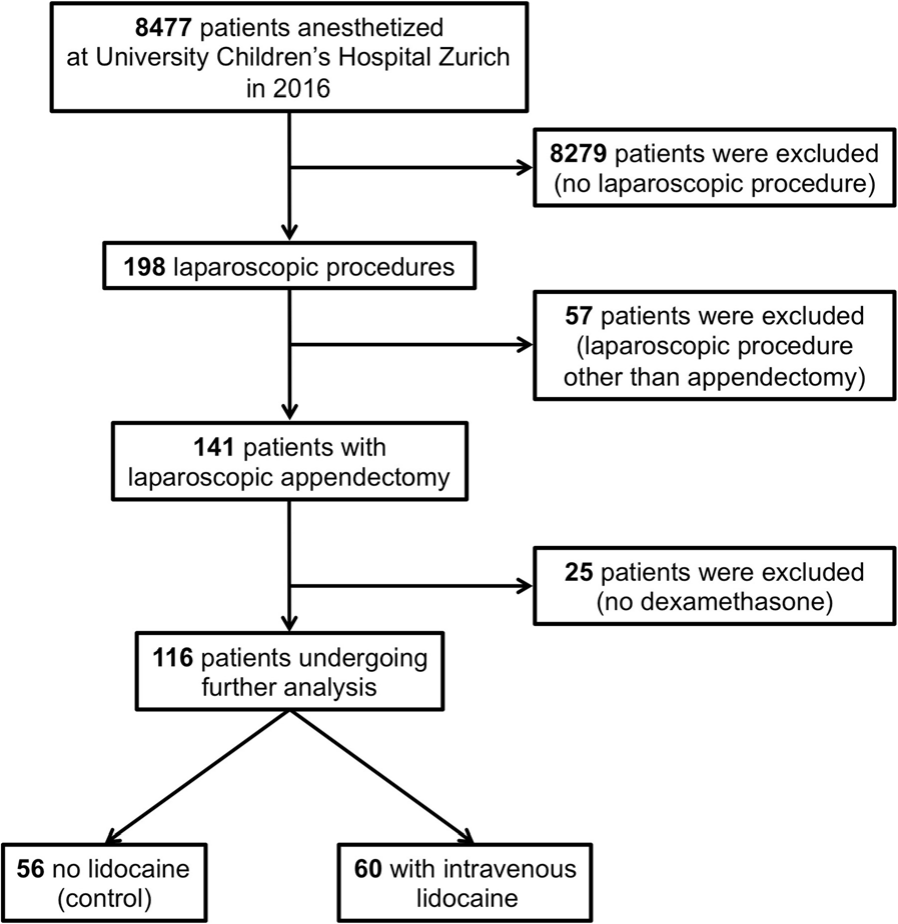
Patient flow chart

With approval of the local ethics committee (Kantonale Ethikkommission, Zurich, Switzerland; study protocol BASEC number: 2016–00188) our anesthesia electronic patient data management system was screened for patients undergoing laparoscopic appendectomy in 2016. To minimize possible confounders, we excluded all patients who had not received dexamethasone for any reason (Fig. 1).

### Data collection

Data were collected from premedication reports and the perioperative patient data management system (MetaVision, Version 5.46.44, iMDsoft, Duesseldorf, Germany) as well as from the patient’s electronic medical records. The primary endpoint was the incidence of any perioperative neurologic (perioral tingling, numbness, seizures) symptoms. Circulatory symptoms (cardio-respiratory collapse or arrest; severe, prolonged or refractory hypotension) as well as apparent behavioral deterioration in the postoperative period such as emergence delirium (ED) documented by PACU or ward staff, were also noted. The assessment of ED is based on clinical criteria of the Pediatric Anesthesia Emergence Delirium (PAED) scale [15]. Besides epidemiological and perioperative data, the duration of continuous lidocaine infusion and the total amount of lidocaine administered were recorded. Additionally, the need for any rescue medication against pain, PONV, the time to first postoperative bowel movement and to hospital discharge after surgery, as well as the incidence of post-operative complications (as classified by the Dindo-Clavien score [16]) were evaluated.

### Management of general anesthesia

The standard premedication drug for children above the age of 6 months is midazolam (0.5 mg/kg body weight (BW), with a maximum of 10 mg orally or rectally) given 20–45 min (depending on the route of application) prior to the transfer to the operating theater. For induction of general anesthesia, opioids (either alfentanil 10–15 mcg/kgBW) or fentanyl (1–3 mcg/kgBW), and propofol (3–5 mg/kgBW) were used, followed by atracurium (1 mg/kgBW) for muscle paralysis. Controlled rapid sequence induction and intubation was performed with gentle mask ventilation prior to endotracheal intubation with a cuffed tube. Anesthesia was maintained either with sevoflurane or a continuous infusion of propofol. Intraoperative analgesia was provided via bolus administration of either alfentanil of fentanyl. Dexamethasone (0.2 mg/kg, max. 8 mg in total), paracetamol and metamizole (15 mg/kgBW each) were administered during anesthesia for initial postoperative pain relief.

All patients received crystalloid fluids (Ringer’s acetate glucose 1%, Bichsel, Switzerland) for maintenance fluid intake. Additional fluids were administered in case of noted hypotension, using a bolus of crystalloids (Ringer-acetate Fresenius i.v., Fresenius Kabi, Switzerland) or colloid fluid solutions (Physiogel® balanced, B. Braun Medical AG, Switzerland). The choice of fluid for the bolus was left to the discretion of the anesthesiologist in charge.

Administration of lidocaine 1% (without preservatives, Hospital Pharmacy, University Children’s Hospital Zurich, Switzerland) was initiated shortly after tracheal intubation in hemodynamically stable patients starting with an intravenous bolus of 1.5 mg/kgBW followed by a continuous infusion at a rate of 1.5 mg/kgBW/h. The latter was continued until the patient was transferred to the ward postoperatively (either directly from the operating theater or from the post-anesthesia care unit (PACU)). Contraindications for the application of lidocaine were a known allergy against any local anesthetic as well as hemodynamic instability.

For wound infiltration, 25% of the maximum bupivacaine dose, i.e. 0.125 ml/kgBW of bupivacaine 0.5% (carbostesin 0.5%, Aspen Pharma, Switzerland) were allowed and were usually administered by the surgeon at the end of surgery.

### Statistical analysis

Categorical data are expressed as the number of patients together with their corresponding percentage of their total group (control = no lidocaine versus lidocaine). Differences between the proportions of qualitative data were assessed with χ^2^ or Fisher’s Exact test where appropriate. Quantitative data were assessed for normal distribution using the Shapiro-Wilk test. Normally distributed data are expressed as mean (SD) and Student’s t-test was used for inter-group comparison. Non-parametric data are reported as median (interquartile range (IQR)) and were compared using a Mann-Whitney U test. Spearman correlation analysis was performed for the evaluation of the relationship between specific factors. A *p*-value < 0.05 was considered to be statistically significant. All analyses were conducted using the SPSS software for Mac (Version 23, IBM Corp, Armonk, NY, USA).

## Results

A total of 8477 patients were anesthetized in our institution in 2016. Of these, 198 patients received anesthesia for laparoscopic procedures and 141 underwent laparoscopic appendectomy. Finally, a total of 116 patients who had received anesthesia for laparoscopic appendectomy and received dexamethasone underwent further analysis (for flowchart see Fig. 1).

Patient characteristics are summarized in Table 1. Patients in the lidocaine group received a mean total of 4.88 (SD 2.76) mg/kgBW lidocaine over 118.2 (57.5) minutes (Table 2). In patients who had received fentanyl only after the induction of anaesthesia, a non-significant difference in the amount of the applied fentanyl could be observed, with patients in the lidocaine group tending to receive less (median of 4.6 vs. 3.7 mcg/kgBW, *p* = 0.12, all Table 2).

**Table 1:** Baseline characteristics of patients

Variable	Control (*n* = 56)	Lidocaine (*n* = 60)	*p*-value
Age [yrs]			
*Mean (SD)*	10.46 (3.63)	10.19 (3.76)	0.69 ^a^
Gender			
*Female/Male (%)*	33 / 23 (58.9 / 41.1)	30 / 30 (50 / 50)	0.335 ^b^
Weight [kg]			
*Median (IQR)*	38.4 (26.5)	35.9 (22.8)	0.3 ^c^
ASA score [number (%)]			
*I / II / III*	39 (69.6) / 15 (26.8) / 2 (3.6)	31 (51.7) / 26 (43.3) / 3 (5.0)	0.140 ^b^
Premedication [number (%)]	50 (89.3)	54 (90.0)	0.9 ^b^

a = Student's t-test, b = Chi-square, c = Mann-Whitney U

**Table 2:** Perioperative characteristics of patients

Variable	Control (*n* = 56)	Lidocaine (*n* = 60)	*p*-value
Duration of surgery [min]			
*Mean (SD)*	79.98 (30.92)	78.50 (34.92)	0.810 ^a^
Amount of lidocaine administered [mg/kg BW]			
*Mean (SD)*	n/a	4.88 (2.76)	
Duration of lidocaine administration [min]			
*Mean (SD)*	n/a	118.2 (57.5)	
Type of anesthesia [number (%)]			0.639 ^d^
*Inhalation*	43	43	
*TIVA*	10	15	
*Balanced*	3	2	
Opioids at induction of anesthesia [number (%)]	55 (98.2)	58 (96.7)	1 ^d^
*Alfentanil*	45 (81.8)	53 (91.4)	0.203 ^d^
*Fentanyl*	9 (16.4)	5 (8.6)	
*Alfentanil + Fentanyl*	1 (1.8)	0 (0)	
Opioids after induction [number (%)]	56 (100.0)	60 (98.3)	
*Alfentanil only*	1 (1.8)	0 (0)	
*Fentanyl only*	40 (71.4)	49 (81.7)	
*Fentanyl intraoperative [mcg/kg BW]*			
*Median (IQR)*	4.63 (2.68)	3.7 (1.95)	0.123 ^c^
*Alfentanil + Fentanyl*	12 (21.4)	3 (5.0)	
*Fentanyl + Remifentanil*	3 (5.4)	6 (10.0)	
*Fentanyl + Morphine*	0 (0)	1 (1.7)	
Perforated appendix [number (%)]	12 (21.4)	20 (33.3)	0.152 ^b^
Crystalloids [ml/kg BW]			
*Mean (SD)*	30.12 (23.11)	27.92 (14.55)	0.539 ^a^
Colloids [number (%)]			
*Total*	1 (1.8)	6 (10.0)	0.115 ^d^
*Perforated / Non-perforated*	1 / 0	4 / 2	1 ^d^
Ephedrine administration [number (%)]			
*Total*	6 (10.7)	13 (21.7)	0.111 ^b^
*Perforated / Non-perforated [number]*	1 / 5	9 / 4	1 ^d^

a = Student's t-test, b = Chi-square, c = Mann-Whitney U, d = Fisher’s Exact; balanced anesthesia = combination of intravenous and volatile anesthetics, SD = standard deviation, IQR = interquartile range, BW = body weight

No difference could be detected in the mean amount of crystalloids applied, but the number of patients receiving colloids or ephedrine was higher in the lidocaine group, although these differences did not reach statistical significance (*p* = 0.115 for colloids, *p* = 0.111 for ephedrine). A Spearman’s correlation analysis evaluating the relationship between a perforated appendix and the intraoperative use of colloids or ephedrine revealed a (weak) correlation for colloids (correlation coefficient 0.25, *p* = 0.007), whereas there was no correlation between the use of ephedrine and a perforated appendix (correlation coefficient − 0.13, *p* = 0.89). The higher number of patients with a perforated appendix in the lidocaine group (12 vs. 20 patients, *p* = 0.152, Table 2), might therefore be a possible explanation for the higher rate of colloid administration in the lidocaine group.

The prescription of postoperative analgesics differed significantly between the two groups, with the lidocaine group requiring less non-opioid analgesics than the control group (*p* = 0.005, Table 3). However, no difference in postoperative opioid requirements could be detected (Table 3).

**Table 3:** Postoperative analgesic requirements in all patients

Variable	Control (*n* = 56)	Lidocaine (*n* = 60)	*p*-value
Postoperative analgesia with non-opioids [number (%)]			0.005* ^c^
*Paracetamol only*	0 (0)	3 (5)	
*Paracetamol + Metamizol*	52 (92.9)	49 (81.7)	
*Paracetamol + Ibuprofen*	1 (1.8)	8 (13.3)	
*Paracetamol + Metamizol + Ibuprofen*	3 (5.4)	0 (0)	
Any opioid in PACU/immediately post-op [number (%)]	23 (41.1)	24 (40)	0.906 ^a^
PCA post-operative [number (%)]			
*Any*	8 (14.3)	13 (21.7)	0.302 ^a^
*Nalbuphine*	3 (5.4)	11 (18.3)	
*Morphine*	5 (8.9)	2 (3.3)	
PCA duration [hrs]			
*Median (IQR)*	54.5 (26)	39 (49)	0.336 ^b^
Any pain rescue medication first 24 h post-op [number (%)]	34 (60.7)	39 (65.0)	0.633 ^a^

a = Chi-square, b = Mann-Whitney U, c = Fisher’s Exact, IQR = interquartile range, significant effects (p<0.05) are marked with “*” and presented in bold and italic font

No neurological disturbances (seizures, numbness, tingling, paresthesias) were noted in any of the patients receiving lidocaine. Instead, there was a trend towards a lower incidence of ED in the lidocaine group (4 vs. 0 patients, *p* = 0.05, Table 4).

**Table 4:** Postoperative outcome measures of all patients

Variable	Control (*n* = 56)	Lidocaine (*n* = 60)	*p*-value
PONV [number (%)]	17 (30.4)	21 (35.0)	0.594 ^b^
Additional PONV-prophylaxis [number (%)]			0.087 ^d^
*None*	7 (12.5)	14 (23.3)	
*Ondansetron*	49 (87.5)	44 (73.3)	
*Ondansetron + Droperidol*	0 (0)	2 (3.3)	
Emergence delirium [number (%)]	4 (7.1)	0 (0)	0.05 ^d^
Time to first bowel movement post-op [hrs]			
*Median (IQR)*	48 (25)	40 (30)	0.05 ^c^
Post-op complications [number (%)]			
*Any*	6 (10.7)	5 (8.3)	0.757 ^d^
*Dindo Score*			
*I*	1 (1.8)	2 (3.3)	
*II*	5 (8.9)	2 (3.3)	
*III*	0 (0)	1 (1.7)	
*IV*	0 (0)	0 (0)	
Discharge after surgery [days]			
*Mean (SD)*	3.75 (2.77)	4.67 (4.52)	0.19 ^a^

a = Student's t-test, b = Chi-square, c = Mann-Whitney U, d = Fisher’s Exact. Dindo Score of postoperative surgical complications [16]: I = alteration of normal postoperative course, no surgical, endoscopic or radiologic intervention needed, allowed medications in grade I: antiemetics, antipyretics, analgetics, diuretics, electrolytes; II = pharmacological treatments other than allowed in grade I, including blood transfusions and total parenteral nutrition; III = complications requiring surgical, endoscopic or radiological intervention, IV = life-threatening complication requiring intensive care unit

None of the patients suffered from cardio-respiratory collapse or arrest, severe, prolonged or refractory hypotension or arrhythmias, especially bradycardia.

A subgroup analysis of all patients with a perforated appendix revealed similar results as observed for the whole study population (Table 5). The median time to the first postoperative bowel movement seemed to be lower in the lidocaine group, although this difference was not statistically significant (median of 59 vs. 39 h, *p* = 0.27).

**Table 5:** Postoperative outcome measures in patients with perforated appendicitis

Variable	Control (*n* = 12)	Lidocaine (*n* = 20)	*p*-value
PONV [number (%)]	4 (33.3)	6 (30.0)	1 ^b^
Emergence delirium [number (%)]	2 (16.7)	0 (0)	0.133 ^b^
Any opioid in PACU/immediately post-op [number (%)]	5 (41.7)	6 (30.0)	0.7 ^b^
PCA post-operative [number (%)]			
*Any*	5 (41.7)	7 (35.0)	0.724 ^b^
*Nalbuphine*	1 (8.3)	6 (30.0)	
*Morphine*	4 (33.3)	1 (5.0)	
PCA duration [hrs]			
*Median (IQR)*	62 (39)	48 (30)	0.43 ^a^
Any pain rescue medication first 24 h post-op [number (%)]	10 (83.3)	12 (60.0)	0.248 ^b^
Post-op complications [number (%)]			
*Any*	5 (41.7)	5 (25.0)	0.438 ^b^
*Dindo Score*			
*I*	1 (8.3)	2 (10.0)	
*II*	4 (33.3)	2 (10.0)	
*III*	0 (0)	1 (5.0)	
*IV*	0 (0)	0 (0)	
Time to first bowel movement post-op [hrs]			
*Median (IQR)*	59 (34)	38.5 (31)	0.27 ^a^
Discharge after surgery [days]			
*Median (IQR)*	7 (6)	6 (4)	0.92 ^a^

a = Mann-Whitney U, b = Fisher’s Exact. Dindo Score of postoperative surgical complications [16]: I = alteration of normal postoperative course, no surgical, endoscopic or radiologic intervention needed, allowed medications in grade I: antiemetics, antipyretics, analgetics, diuretics, electrolytes; II = pharmacological treatments other than allowed in grade I, including blood transfusions and total parenteral nutrition; III = complications requiring surgical, endoscopic or radiological intervention, IV = life-threatening complication requiring intensive care unit

## Discussion

This retrospective study analyzed perioperative factors associated with the systemic use of lidocaine in pediatric patients. Based on our knowledge, it includes the highest number of pediatric patients assessed for the effects of the drug after administering it intra- and postoperatively. On July 1st, 2016, perioperative administration of intravenous lidocaine was added to our standard of care for all laparoscopic procedures at the University Children’s Hospital Zurich, Switzerland. This decision was based on the strong evidence for potential beneficial effects, for example regarding postoperative pain control or PONV. As appendectomy was the most common laparoscopic procedure in our institution and in order to minimize possible confounders, only data from patients undergoing this particular surgery were analyzed.

The main finding was that neither signs of local anesthetic systemic toxicity nor circulatory instability or disturbances were noted in any of the patients investigated.

Local anesthetic systemic toxicity (LAST) has been discussed and studied at large in recent years and guidelines have been implemented for its treatment [17]. However, most of the previously published analyses have examined the incidence of LAST after peripheral nerve blocks and not after intravenous use [18] and only scarce evidence exists regarding safety and potentially beneficial effects of intravenous lidocaine in children. In a study of 91 patients undergoing procedural sedation no benefit of pre-emptive lidocaine administration (0.5 mg/kgBW bolus) on the injection pain due to propofol could be detected [19]. Two small studies have examined safety aspects of intravenous lidocaine for the treatment of chronic pain in adolescents [20, 21]: Mooney and colleagues reported a small series of 15 patients aged 12–19 years, who had received a total of 58 lidocaine infusions with an infusion rate of up to 60 μg/kg/min (i.e. 3.6 mg/kgBW/h) with no initial bolus. The most common side effects reported were “dizziness” and sensations termed as “numbness” or “tingling”, which occurred in 16 and 10%, respectively. However, 79% did not experience any side effects at all [20]. The other recent study retrospectively analyzed 4 patients aged 8–18 years undergoing several treatments with intravenous lidocaine for opioid-refractory cancer pain: Here the authors reported an incidence of side effects such as visual changes or hallucinations, as well as paresthesias during 35% of the treatments [21]. However, with a median infusion rate of 30 μg/kg/min (i.e. 1.8 mg/kgBW/h), the rate in these patients was still higher than in our institution (1.5 mg/kgBW/h). Due to the fact that it is impossible to detect most of these symptoms with the patient held under general anesthesia, the current study might of course fail in detecting them.

The dosage of 1.5 mg/kgBW bolus as used in the current study has been chosen after it has been reported as the most common dose used in 64% of trials included in a recent Cochrane analysis [14]. According to the same Cochrane analysis, the continuous infusion rate of 1.5 mg/kgBW/h might be rather low compared to other studies but has still been reported to be effective in combination with a reasonable risk of toxicity [14]. However, it might of course be difficult to predict toxic effects only based on weight adjusted administration local anesthetics due to their cumulative effects [22].

No neurologic side effects, such as visual disturbances, tingling or paresthesia, have been observed by the PACU staff, even in patients fully awake again after emergence from general anesthesia, while still under continuous lidocaine infusion or after being transferred to the ward shortly after the cessation of the lidocaine infusion.

The new and unexpected finding in the current study was the trend towards a reduction of the incidence of emergence delirium (ED) in patients receiving lidocaine. ED is a common event in pediatric anesthesia with an incidence varying between 10 and 67% [23]. The prevalence of ED has risen with the more widespread use of sevoflurane (and desflurane) [24], however, the pathophysiologic changes leading to this phenomenon are not completely understood. In adults, endothelial dysfunction due to the release of inflammatory cytokines is a common perioperative event and a crucial factor influencing the incidence of delirium [25, 26]. Amide-local anesthetics have been demonstrated to be able to preserve endothelial barrier function upon an inflammatory stimulus [5, 6] and might therefore also potentially be able to influence pathophysiologic events leading to neurologic disturbances after surgery.

Postoperative pain might not also be a trigger for ED, but might it also be difficult to be discriminated from the latter [27] and it is known that pain is a common problem after paediatric laparoscopic appendectomy, with 33% of children suffering from substantial pain on the day of surgery and still 20% on postoperative day 1 in a recent retrospective study of 186 patients [28]. Patients in the current study received a well-established, pre-emptive analgesic regimen, which might also impede the detection of a possible effect of lidocaine in this setting. However, we could detect a significantly lower prescription rate of non-opioid analgesics in the lidocaine group, whereas the number of patients requiring any form of opioid rescue medication did not differ between the groups.

A suggested reduction of PONV by lidocaine might also be difficult to reproduce in this specific patient population, as patients with an acute appendicitis already show signs of severe inflammation as well as nausea and vomiting preoperatively caused by intestinal disturbances [29, 30]. In addition, all patients in the current study received at least dexamethasone as an anti-emetic prophylaxis (with 82% receiving a second drug as well), which might also make it even more difficult to detect any lidocaine-associated benefits regarding PONV and pain [31].

The time to first bowel movement was reduced in the lidocaine group by 17% overall and by 33% in patients with a perforated appendicitis, although the detected differences did not reach statistical significance. However, these findings are in accordance with a recent Cochrane analysis suggesting a beneficial effect of lidocaine via an enhanced gastrointestinal recovery in patients undergoing abdominal surgery [14].

There are several limitations of this study, which were mainly due to its retrospective character. Overall, interpretation of the reported data should be made with caution, especially due to the fact that this study compares patients with lidocaine administration to a historic group of patients without. Additionally, the number of patients is rather small, which might explain the fact that several observed differences did not reach statistical significance. Additionally, the limited data on postoperative pain intensity and quality makes it difficult to conclude a potential beneficial effect of lidocaine on postoperative analgesia in this specific patient population.

## Conclusions

In this retrospective data analysis in a limited number of pediatric patients undergoing general anesthesia for laparoscopic appendectomy, the perioperative continuous administration of intravenous lidocaine did not lead to any adverse effects. However, beneficial effects should further be assessed in future randomized controlled clinical trials in combination with a potentially more detailed analysis regarding the effects as well as the pharmacokinetic properties of lidocaine in the pediatric population.

## Abbreviations

BW: body weight
ED: emergence delirium
IQR: interquartile range
PACU: post-anesthesia care unit
PCA: patient-controlled analgesia
SD: standard deviation
